# Supplementary material for: Dietary restriction fails to extend lifespan of Drosophila model of Werner syndrome
Source: G3 (Bethesda). 2024 Mar 16;14(5):jkae056. doi: 10.1093/g3journal/jkae056 (PMC11075538; doi:10.1093/g3journal/jkae056)
Supplement: jkae056_Supplementary_Data [file jkae056_supplementary_data.zip › Table_S1_and_Figure_S1_G3-2024-404820.docx]

**Supplementary Information for**

**Dietary Restriction Fails to Extend Lifespan of *Drosophila* Model of Werner Syndrome**

Eileen Sember, Ranga Chennakesavula, Breanna Beard, Mubaraq Opoola, Dae-Sung Hwangbo^*^

Department of Biology, University of Louisville, Louisville, KY, USA

***Correspondence:**

Dae-Sung Hwangbo, Ph.D.

Department of Biology, University of Louisville

139 Life Sciences Bldg., Louisville, KY, 40292, USA.

Email address: [ds.hwangbo@louisville.edu](mailto:ds.hwangbo@louisville.edu)

Tel: 1-502-852-5937

This file includes Supplementary table (Table S1) and figure (Figure S1)

Other supplementary materials for this manuscript include supplementary files S1-S4 (excel files)

| **g/100mL of diet** | **Bloomington Formula**  **(Nutri-Fly^®^)** | **1Y**  **(1% yeast, malnutrition)** | **5Y**  **(5% yeast, DR)** | **20Y**  **(20% yeast, Con)** |
| --- | --- | --- | --- | --- |
| **Calories** | 55.17 | 23.48 | 38.95 | 97.00 |
| **Fiber** | 1.49 | 1.40 | 2.20 | 5.20 |
| **Sugars** | 1.96 | 5.00 | 5.00 | 5.00 |
| **Protein** | 1.83 | 0.53 | 2.66 | 10.61 |
| **Fat** | 0.48 | 0.04 | 0.04 | 0.04 |
| **Carbohydrates** | 12.34 | 0.04 | 8.35 | 14.80 |

**Table S1.** **Nutritional contents of the Bloomington formula and the SY diets**.

The nutritional values of the three main diets used in this study were compared to those of the Bloomington formula (Nutri-Fly^®^, Genesee Scientific, Cat#: 66-113) using the Drosophila Dietary Composition Calculator (DDCC) [1]. The DDCC is publicly accessible at the following link: <https://www.brodericklab.com/DDCC.php>


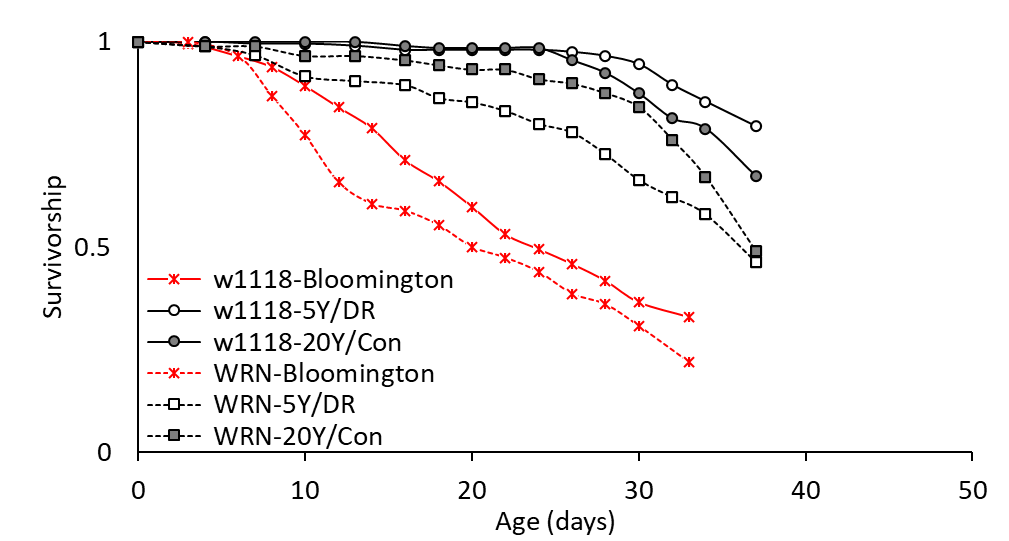


**Figure S1.** **Lifespan comparison of *w^1118^* and *WRNexo^Δ^* mutants on the Bloomington formula diet and SY diets**.

Female flies of ~72-hour cohorts were collected. They were allowed one day of post-eclosion mating before being set up for lifespan studies on each diet. Six to eight replicate vials, each containing up to 25 flies, were set up for each diet. Bloomington: The Bloomington formula (Nutri-Fly®, Genesee Scientific, Cat#: 66-113), 5Y/DR: 5% Yeast and 5% Sucrose, 20Y/Con: 20% Yeast and 5% Sucrose. Sample size at the start: ~ 200 flies in 8 replicates for *w^1118^* and 88 ~ 114 flies in 5 ~ 7 replicates for *WRNexo* mutants.

**References**

1. Lesperance, D.N.A. and N.A. Broderick, *Meta-analysis of Diets Used in Drosophila Microbiome Research and Introduction of the Drosophila Dietary Composition Calculator (DDCC).* G3 (Bethesda), 2020. **10**(7): p. 2207-2211.
